# MICE or NICE? An economic evaluation of clinical decision rules in the diagnosis of heart failure in primary care^☆^^☆☆^

**DOI:** 10.1016/j.ijcard.2017.02.149

**Published:** 2017-08-15

**Authors:** Mark Monahan, Pelham Barton, Clare J Taylor, Andrea K Roalfe, F.D. Richard Hobbs, REFER investigators, Martin Cowie, Russell Davis, Jon Deeks, Jonathan Mant, Deborah McCahon, Theresa McDonagh, George Sutton, Lynda Tait

**Affiliations:** a Institute of Applied Health Research, University of Birmingham, Edgbaston, Birmingham B15 2TT, United Kingdom; b Nuffield Department of Primary Care Health Sciences, Radcliffe Primary Care Building, Radcliffe Observatory Quarter, University of Oxford, OX2 6GG, United Kingdom; c Faculty of Medicine, National Heart and Lung Institute, Imperial College London, South Kensington Campus, London SW7 2AZ, United Kingdom; d Department of Cardiology, Sandwell and West Birmingham Hospitals, Lyndon, West Bromwich, West Midlands B71 4HJ, United Kingdom; e Department of Public Health & Primary Care, University of Cambridge, Strangeways Research Laboratory, Wort's Causeway, Cambridge CB1 8RN, United Kingdom; f Department of Cardiology, King's College Hospital, Denmark Hill, London SE5 9RS, United Kingdom; g School of Health Sciences, University of Nottingham, B Floor, South Block Link, Queen's Medical Centre, Nottingham NG7 2HA, United Kingdom

**Keywords:** Medical economics, Economic model, Cost benefit analysis, Natriuretic peptide, General practice

## Abstract

**Background:**

Detection and treatment of heart failure (HF) can improve quality of life and reduce premature mortality. However, symptoms such as breathlessness are common in primary care, have a variety of causes and not all patients require cardiac imaging. In systems where healthcare resources are limited, ensuring those patients who are likely to have HF undergo appropriate and timely investigation is vital.

**Design:**

A decision tree was developed to assess the cost-effectiveness of using the MICE (Male, Infarction, Crepitations, Edema) decision rule compared to other diagnostic strategies to identify HF patients presenting to primary care.

**Methods:**

Data from REFER (REFer for EchocaRdiogram), a HF diagnostic accuracy study, was used to determine which patients received the correct diagnosis decision. The model adopted a UK National Health Service (NHS) perspective.

**Results:**

The current recommended National Institute for Health and Care Excellence (NICE) guidelines for identifying patients with HF was the most cost-effective option with a cost of £4400 per quality adjusted life year (QALY) gained compared to a “do nothing” strategy. That is, patients presenting with symptoms suggestive of HF should be referred straight for echocardiography if they had a history of myocardial infarction or if their NT-proBNP level was ≥ 400 pg/ml. The MICE rule was more expensive and less effective than the other comparators. Base-case results were robust to sensitivity analyses.

**Conclusions:**

This represents the first cost-utility analysis comparing HF diagnostic strategies for symptomatic patients. Current guidelines in England were the most cost-effective option for identifying patients for confirmatory HF diagnosis. The low number of HF with Reduced Ejection Fraction patients (12%) in the REFER patient population limited the benefits of early detection.

## 1 Introduction

Heart failure (HF) is a common clinical condition which is associated with major impact for patients and high costs for health systems, but is not easy to diagnose accurately or early in primary care [1], [2], [3].

The main symptoms suggestive of HF are shortness of breath, tiredness and swollen ankles but these complaints are common in primary care and most patients presenting with them will not have HF [4]. Furthermore, referring all symptomatic patients on for confirmatory investigations, such as echocardiography, is expensive. However, early detection of HF is important since evidence-based therapies can substantially improve quality of life, reduce premature mortality, and reduce avoidable hospital admissions [5], [6]. The optimal cost-effective strategy for diagnosing HF patients at primary care level is not known.

There is a paucity of data on diagnostic strategies in patients presenting in primary care with symptoms suggestive of HF where the population are rigorously phenotyped for HF. Economic analyses of diagnostic triage for this patient population are rarer still, but the REFER (REFer for EchocaRdiogram) study provides appropriate data to undertake an economic evaluation.

REFER [7] was a prospective, observational, diagnostic validation study of the MICE (Male, Infarction, Crepitations, Edema) clinical decision rule–with natriuretic peptide testing–for diagnosing HF in primary care. The full methods for the study have been previously published elsewhere [8]. The clinical decision rule was developed from an Individual Patient Database meta-analysis of epidemiological studies of HF screening in primary care [9].

Briefly, primary care patients aged 55 years or over presenting to their GP with symptoms suggestive of HF were recruited across 28 Central England practices in the UK. All consenting patients underwent a full clinical assessment, which included a NT-pro B-type natriuretic peptide (NT-proBNP) test, an echocardiogram and quality of life questionnaire, at a research clinic within one week of recruitment. Follow-up quality of life and resource use questionnaires were mailed to the patients at six and twelve months after attending the clinic.

The 304 patients included in the REFER study had the following characteristics: mean age of 73.9 years, White ethnicity (70.4%), male (40.8%), history of myocardial infarction (11.2%), basal crepitations (5.3%), ankle oedema (81.6%), lethargy (74.3%) and had a median NT-proBNP of 214 pg/ml (IQR 79 pg/ml–494 pg/ml).

The diagnosis of ‘heart failure’ or ‘no heart failure’ was determined by an expert panel of cardiologists using the European Society of Cardiology (ESC) 2012 definition [5]. Clinical information, including the variables of the MICE rule and NT-proBNP level, was presented in stages to quantify any incorporation bias.

The aim of this study was to assess the cost-effectiveness of using the MICE clinical decision rule in HF diagnosis in Primary Care from a National Health Service (NHS) and Personal Social Services perspective. To do so, a decision tree was developed comparing different diagnostic strategies against the clinical decision rule. The economic analysis utilized the REFER dataset to determine which symptomatic patients received the correct diagnosis decision. The economic evaluation took a lifetime horizon and all costs and outcomes were discounted at an annual rate of 3.5% as recommended by the National Institute of Health and Care Excellence (NICE) [10].

## 2 Methods

The six comparators are described below. Since the MICE rule has lower and upper cut-offs for the NT-proBNP referral levels, we treated them as two different diagnostic comparators: MICE upper cut-off levels and MICE lower cut-off levels. Strategies differed in terms of immediate actions. All patients with true HF who were not referred at this stage were assumed to return six months later and such patients will be referred immediately for echocardiography.

### 2.1 Economic evaluation diagnostic pathways

#### 2.1.1 MICE clinical decision rule

The MICE clinical decision rule states a patient presenting with HF symptoms at the GP will be referred straight for echocardiography if the patient has either a history of myocardial infarction (MI), or basal crepitations, or is a male with ankle oedema. Otherwise a NT-proBNP test is carried out and the patient is referred straight for echocardiography if the test results are above one of three cut-offs set by gender/symptoms recorded in the clinical rule (where the upper MICE NT-proBNP cut-off levels are in parentheses):

• A female patient without ankle oedema should be referred if NT-proBNP is ≥ 620 pg/ml (1060 pg/ml),or
• A male patient without ankle oedema should be referred if NT-proBNP is ≥ 390 pg/ml (660 pg/ml),or
• A female patient with ankle oedema should be referred if NT-proBNP is ≥ 190 pg/ml (520 pg/ml)

### 2.2 NICE recommended strategy

NICE guidelines for the management of chronic HF^6^ suggest that a patient presenting with symptoms suggestive of HF should be referred straight for echocardiography if they have a history of MI. Otherwise a NT-proBNP test should be carried out and patient referred for an echocardiograph if the NT-proBNP level is ≥ 400 pg/ml.

### 2.3 Echo all strategy

With the echo all strategy, all patients presenting with HF symptoms at the GP will be referred straight for echocardiography. We make a simplifying assumption that there will be no problems with access to echocardiography.

### 2.4 NT-proBNP 125 strategy

With the NT-proBNP 125 strategy, all patients presenting with HF symptoms at the GP will have a NT-proBNP test carried out and the patient is referred for echocardiography if their NT-proBNP level is ≥ 125 pg/ml.

### 2.5 Do nothing strategy

With the do nothing strategy, no patients presenting with HF symptoms at the GP will be referred straight for echocardiography nor undergo a NT-proBNP test. This option was added in for completeness.

**Fig. 1:**
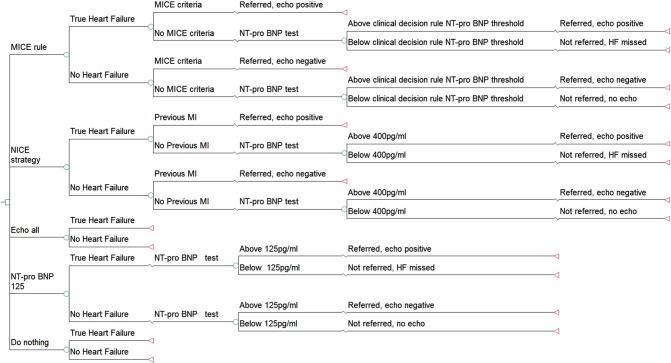
Decision tree of the different diagnostic strategies for patients presenting with heart failure symptoms in primary care.

A decision tree, presented in TreeAgePro 2014 (TreeAge Software, Williamstown, MA) and developed in Excel, was structured to represent the various diagnostic strategies (Fig. 1). A decision tree was appropriate here as the comparators diverge with regard to immediate actions. Branch probabilities were estimated from the REFER dataset. To ensure correct representation of the statistical uncertainty in the model, patients were categorised into groups (Appendix Table 1), on the principle that two patients would be in the same group if and only if they follow the same pathway in all strategies considered. For example, consider four patients of the same sex and with the same clinical signs, with no previous MI, but with NT-proBNP at 110 pg/ml (A), 220 pg/ml (B), 330 pg/ml (C), and 440 pg/ml (D). Under the “NT-proBNP 125” strategy, patients B, C and D would be referred for echocardiography, but A would not, while under the NICE strategy, only patient D would be referred. However, no strategy in the model has a cut-off between 220 pg/ml and 330 pg/ml, so patients B and C can be in the same group.

The methodology behind the benefits of each strategy was drawn largely from a previous HF Health Technology Assessment (HTA) report [9]. We assumed a confirmed HF diagnosis leads to patients being initiated on HF drug treatment if they have HF with Reduced Ejection Fraction (HFREF). Current trials do not suggest a survival advantage of angiotensin converting-enzyme inhibitors (ACEIs) or beta-blockers for HF patients with preserved ejection fraction (HFPEF) [11]. Thus, the benefits from early detection of HF were weighted by the number of HF patients in the population (12%) that have a reduced ejection fraction.

The type of HF drug and dose was obtained from a cohort study of patients treated for HF in primary care; 36.6% of patients were treated with beta-blockers, 58.9% of patients were treated with ACEIs, and 13.4% of patients were treated with angiotensin receptor blockers (ARBs) [12]. Patients can be on more than one HF drug therapy; to determine the proportion of patients on ACEI but not on beta-blockers (not reported), we took a simple average of upper bound and lower bound range of the possible values (36.3%).

HF treatment initiation due to correct early detection was assumed to have a survival benefit for the HF patient versus a missed HF diagnosis. A missed HF diagnosis was assumed to delay the diagnosis by six months. Delayed diagnosis patients were assumed to incur a further GP visit and an echocardiogram to confirm the diagnosis. After six months, the patient was put on treatment and thereafter has the same survival probability as someone who was already on treatment.

We took the survival data from diagnosis of HF in patients in the Framingham heart study [13] to be the patient's prognosis without drug therapy. A meta-analysis indicated that beta-blockers versus placebo gave a hazard ratio of 0.73 (95% confidence interval 0.67–0.80) for all-cause mortality, 95% of these beta-blockers patients were also on ACEI/ARBs [14]. The results of a systematic review showed that the mortality odds ratio for patients taking ACEI compared to a placebo was 0.80 (95% confidence interval 0.74–0.87) [15]. A Cochrane review found no significant effect of ARBs on mortality and, therefore, we make a conservative assumption that patients on ARBs only have the same survival rate as untreated patients [16].

Survival data and drug efficacies were used to plot gender-specific survival curves for the untreated patients and patients on different drug therapies (assuming no temporal changes in drug efficacy). The curves were extended beyond the ten year survival data via linear extrapolation to achieve a lifetime horizon. Patients who were put on treatment earlier due to correct detection (Treat early) and patients with a delayed diagnosis (Treat late) were weighted by the proportion of patients on the different drug therapies. Survival benefit due to early treatment was the area between the treated early and treated late curves.

Patients with HF in the REFER study that answered the six months follow-up questionnaire, scored with UK tariffs [17], gave an average EuroQol 5 dimensions (EQ-5D) score of 0.615 (standard deviation of 0.31). Discounted quality adjusted life year gain can be estimated from the product of the EQ-5D and the discounted life years gained.

Early detection of HF also has the benefit of a reduction in hospitalizations. Patients on beta-blockers versus placebo have a hazard ratio of 0.71 (95% CI 0.65–0.77) for first HF related hospital admission [14]. Similarly, treatment with ACEIs reduces the likelihood of a hospital admission with an odds ratio of 0.67 (95% CI 0.61–0.74) [15].

For model purposes, we were interested in the increased rate of admission into hospital for an untreated population due to a six month delay in a HF diagnosis. Firstly, we take a previously calculated probability of admission if treated of 41.3% [9]. Using the odds ratio of hospital admission with ACEI treatment, the rate of admission within six months if untreated is 0.62. Assuming a constant hazard ratio for treatment with beta-blockers over the six months, the rate of admission within six months if untreated is 71.2%. A simple average was taken to calculate the estimated rate of admission with six months if untreated (66.4%). Thus, the increased number of hospitalization cases if untreated is 25.1%. This was then weighted by the number of HFREF patients in the population as only these patients receive prognostic benefit from treatment.

Costs were inflated to 2013/2014 prices using the hospital & community health service (HCHS) pay and prices index where applicable [18]. The cost of a General Practitioner appointment (£46) was taken from Unit Costs [18]. The cost of an avoidable HF hospital admission was calculated from weighted average reference costs of HF admissions (£2107) [19]. An echocardiography referral cost was taken from the Payment by Results mandatory tariff [20], consisting of a simple echocardiogram and a consultant-led outpatient first attendance (totalling £241). The cost of a NT-proBNP test (£30) was taken from a NICE costing report [21].

The early treatment HF drug cost was estimated from the resource usage combined with prices from the British National Formulary (BNF) [22]. Since the typical drug dose for a HF patient is variable, we pragmatically assumed that same percentage of patients reach the target dose–as defined by the 2005 ESC guidelines in their study–and the remainder achieve half the target dose. Where the drug listed was not recommended by the 2012 ESC guidelines [5], the target dose indication for HF in the BNF was used. For the base-case analysis, the cost of the generic drugs for the first six months was used (£10); the cost of the equivalent therapy with branded drugs was substituted for the sensitivity analysis (£30).

Results were presented as the costs and effects of each strategy and ordered by increasing cost. Effectiveness was measured in quality adjusted life years (QALYs). The incremental analysis was designed to generate the cost per additional QALY gained for using one diagnostic strategy over another. Cost-effectiveness was assessed in relation to the lower NICE threshold of £20,000 per QALY gained [23]. More costly and less effective options were excluded from consideration (dominated). Likewise options that suffer from extended dominance were removed from consideration. Extended dominance occurs when an option would be dominated compared to a mixed option of two other strategies.

Sensitivity analysis was performed to assess model robustness. For the deterministic sensitivity analysis, the following scenarios were explored:

1. Doubling and halving the cost of a NT-proBNP test.
2. Altering the drug efficacies to their lower and upper confidence intervals respectively.
3. Substituting in branded drug therapy prices for generic drug therapy prices.
4. Increasing the proportion of HFREF patients from 12% to 24%, 50%, and 100% respectively.

For the probabilistic sensitivity analysis, distributions were attached to the clinical parameters, drug efficacies, and HF utility (Table 1). The model was run for 10,000 iterations and the results presented as a cost-effectiveness acceptability frontier (CEAF). The CEAF shows, across a range of different cost-effectiveness thresholds, the uncertainty associated with the optimal diagnostic option.

**Table 1:** Parameter distributions for probabilistic sensitivity analysis.

Parameters	Distribution	Parameter estimates^a^	Deterministic value^b^	Source
Patients true diagnosis	Beta	α = 104	34.2%	REFER dataset [7]
		β = 200		
Heart failure utility (EuroQol-5 dimensions)	Beta	α = 0.95	0.62	REFER dataset [7]
		β = 0.59		
Beta-blocker effect on mortality	Log normal	μ = − 0.31	0.73	Kotecha and colleagues [14]
		σ = 0.05		
Beta-blocker effect on hospitalization risk	Log normal	μ = − 0.34	0.71	Kotecha and colleagues [14]
		σ = 0.04		
ACEI effect on mortality	Log normal	μ = − 0.22	0.80	Flather and colleagues [15]
		σ = 0.04		
ACEI effect on hospitalization risk	Log normal	μ = − 0.40	0.67	Flather and colleagues [15]
		σ = 0.05		
Patients on each drug therapy (beta-blockers, ACEI, other)	Dirichlet	(α_1_,α_2_,α_3_) = (3403,3378,1908)	(36.5%,36.3%,6.1%)	Calvert and colleagues [12]

ACEI, angiotensin converting-enzyme inhibitors.

a For the lognormal distributions, μ is the mean and σ is the standard deviation of the underlying normal distribution which gives the logarithm of the model parameter.

b The deterministic value is the mean except in the case of the lognormal distributions where the median is given.

Positive count data in patient groups formed the parameters for the Dirichlet distribution (Appendix Table 1). On each replication a vector of probabilities was sampled from the appropriate distribution. Where there was no count data (no patients) in a group, no distribution was attached. Treating the probability as fixed for these empty patient groups will slightly underestimate the uncertainty in the model rather than the alternative of adding an occurrence and positively biasing the amount of occurrences [24].

## 3 Results

Costs and effectiveness of each strategy are shown in Table 2. The clinical decision rules of MICE were dominated by the other strategies. Given a willingness to pay (WTP) threshold of £20,000/QALY, only the NICE strategy was cost-effective.

**Table 2:** Base-case results.

Strategy	Costs (£)	QALY gain compared to “do nothing”	Cost per QALY	Proportion of true HF detected	Proportion of not HF ruled out
Do nothing	119	–		0.00%	100.00%
NICE strategy	142	0.0051	£4400	78.85%	63.50%
MICE upper cut-off	167	0.0050	(Dominated)	81.73%	84.00%
MICE lower cut-off	191	0.0057	(Extended dominance)	90.38%	45.50%
NT-proBNP 125	196	0.0059	£69,000	94.23%	49.00%
Echo all	241	0.0063	£125,100	100.00%	0.00%

QALY, quality adjusted life year; HF, heart failure; NICE, National Institute for Health and Care Excellence; cost per QALY rounded to the nearest multiple of £100/QALY.

Sensitivity analyses (Table 3) showed that NICE strategy remained the most cost-effective option for each scenario except where the proportion of HFREF changed to 50% and above. When the proportion of HFREF patients was 50%, the NT-proBNP 125 strategy became cost-effective, and when this proportion reaches 100%, it became cost-effective to refer all patients for immediate echocardiography.

**Table 3:** Deterministic sensitivity analysis scenario results.

	NICE strategy versus do nothing	NT-proBNP 125 versus NICE strategy	Echo all versus NT-proBNP 125
Scenario^a^	Cost per additional quality adjusted life year (QALY)		
Base case results	£4400	£69,000	£125,100
Double NT-proBNP test cost	£9600	£73,300	£42,100
Half NT-proBNP cost	£1800	£66,800	£166,600
Branded drug price therapy	£9700	£68,900	£125,200
Higher drug efficacy for mortality	£3300	£52,000	£94,200
Lower drug efficacy for mortality	£6600	£104,200	£189,100
Proportion of HFREF patients doubled to 24%	£600	£32,900	£60,900
Proportion of HFREF patients increased to 50%	Dominates do nothing	£13,300	£26,400
Proportion of HFREF patients increased to 100%	Dominates do nothing	£5000	£11,600

NICE, National Institute for Health and Care Excellence; HFREF, Heart Failure with Reduced Ejection Fraction; cost per QALY rounded to the nearest multiple of £100/QALY.

a The MICE cut-off options were excluded from the table as they remain dominated in each scenario.

**Fig. 2:**
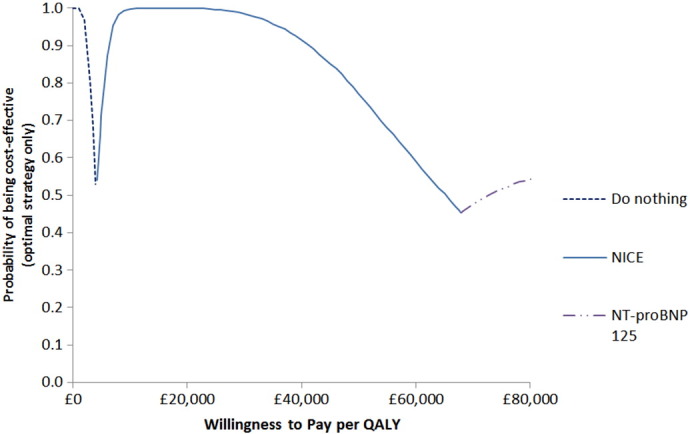
Cost-effectiveness acceptability frontier showing the optimal diagnostic strategy across a range of willingness to pay thresholds.

Fig. 2 illustrates the overall uncertainty related to the optimal decision across a range of plausible WTP values, where WTP was measured in cost per additional QALY. At £20,000/QALY, the likelihood of the NICE strategy being the optimal option (i.e. highest Net Monetary Benefit) is 99.9%. As the WTP threshold increases beyond £68,000, the NT-proBNP 125 option becomes more likely to be the optimal option.

## 4 Discussion

Results indicate that, for the REFER population, current NICE guidelines on diagnosing HF represent the most cost-effective strategy compared to the MICE decision rules and other diagnostic strategies. That is, patients presenting with symptoms suggestive of HF should be referred straight for echocardiography if they have a history of MI or if their NT-proBNP level is ≥ 400 pg/ml. The MICE decision rules were not cost-effective due to dominance by the other strategies. The cost-effectiveness results were robust to deterministic and probabilistic sensitivity analysis.

Modest effectiveness gains for each strategy can be largely explained by the REFER study patient characteristics. The modelled benefits of early detection of HF and the subsequent prognostic window of improvement (treated early versus treated late) were limited since only 12% of the REFER HF participants were HFREF patients. We modelled no benefits from earlier detection in the 88% of the REFER HF population detected with non-HFREF. This is an appropriate conservative assessment but it is unlikely that these patients will derive zero benefit from detection. Although drug trials in HFPEF have failed to prove prognostic benefit, there is still a need for diagnosis to allow clinicians to explain and manage patient symptoms, and for ongoing research to find novel approaches to HFPEF treatment.

Increasing the proportion of HREF patients raised the total QALYs of each diagnostic strategy due to the higher rewards of a correct early detection. Most population surveys suggest that approximately half of HF patients suffer HFREF, in contrast to the 12% detected in REFER, and in this sensitivity scenario the dominant cost-effective strategy is the reduced NT-proBNP cut-off for referral for echocardiography of 125 pg/ml rather than the NICE level of 400 pg/ml. Echo all strategy became the most cost-effective option when we assume all the HF patients will receive prognostic benefit from treatment. However, it must be acknowledged that there may be practical barriers to the implementation of such a strategy. Delays to echocardiograms may potentially offset the advantage of early detection and referring all patients for an echocardiogram will put pressure on local diagnostic services.

Since NT-proBNP came into more general use in the healthcare system, the costs per test may have dropped so the cost-effectiveness of natriuretic peptide testing may be underestimated here. Halving the cost of a NT-proBNP test made the NT-proBNP strategies more cost-effective compared with the ‘echo all’ and the ‘do nothing’ strategies.

HF therapy drug mix related to a 2009 publication from older data [12]. As it is likely that there are higher drug rates nowadays, the assumed proportion of patients on ACEIs and beta-blockers may be underestimated so the benefit of early diagnosis may be greater. Since the clinical study has been carried out, guidelines on the diagnosis and treatment of heart failure have been updated; [25] hence diagnosis of HF is according to 2012 definitions and HF with Mid-Range Ejection Fraction was not considered as a third group as in the 2016 guidelines.

This is the first cost utility analysis to compare a representative range of strategies for diagnosis in this patient population, making comparison to other studies difficult. Previous studies [26], [27] have looked at the costs of using NT-proBNP as a means to rule out HF and to reduce the levels of echocardiography referrals but these cannot address the question of an optimal cost-effective diagnostic strategy and also did not place a cost on a missed HF diagnosis.

The main strength of this analysis is that the diagnostic accuracy of the various strategies tested was calculated based on a consistent primary dataset. This was reinforced by the process used in the clinical study to develop an appropriate gold standard against which to compare imperfect diagnostic strategies. The main limitations are that the REFER dataset may not be typical of patients in other geographic areas, and the need for assumptions in projecting the lifetime costs and outcomes from correct diagnosis. The low prevalence of HFREF in the REFER patient population may be due to patients with HFREF being more likely to present themselves directly to secondary care rather than primary care [7]. The lack of representation of HFREF patients compared with other studies may represent a selection bias affecting the final results.

Overall, this analysis provides evidence based on primary data that the current strategy recommended by NICE is appropriate. However, based on sensitivity analyses, as the proportion of HFREF increases from the 12% seen in REFER, it becomes more cost-effective to change the NICE NT-proBNP threshold from 400 pg/ml to 125 pg/ml.

## Conflicts of interest

All authors have completed the ICMJE uniform disclosure form at http://www.icmje.org/coi_disclosure.pdf and declare: Dr. Hobbs reports grants from Roche diagnostics, outside the submitted work. All other authors declare no conflicts of interest.

## REFER study investigators

Dr. Pelham Barton, University of Birmingham; Prof Martin Cowie, National Heart and Lung Institute, Imperial College, London; Dr. Russell Davis, Sandwell and West Birmingham Hospitals NHS Trust; Prof Jon Deeks, University of Birmingham; Prof Richard Hobbs, University of Oxford (PI); Mrs Rachel Iles, University of Birmingham; Prof Jonathan Mant, University of Cambridge; Dr. Deborah McCahon, University of Birmingham; Prof Theresa McDonagh, King's College, London; Mrs Andrea Roalfe, University of Birmingham; Dr. George Sutton, Imperial College, London; Dr. Lynda Tait, University of Nottingham; Dr. Clare J Taylor, University of Oxford.

## Trial registration

ISRCTN17635379.

## Author contributions

MM conducted the health economic evaluation. PB was the main supervisor of this analysis. RH, LT, AR, MC and JM conceived of and designed the REFER study. LT, AR, JM, MC, JD, PB, CT and RH were grant applicants. LT was senior study co-ordinator for most of the study. DM was study co-ordinator for part of the study. RI was study co-ordinator throughout the study. AR was the study statistician. PB was health economist on the study. GS, MC, RD and TM were cardiologists on the expert panel, RD replacing GS towards the end of the study. CT and RH provided clinical input throughout the study. MM drafted the first manuscript. PB, CT and RH contributed to the writing of the manuscript. All authors read and approved the final manuscript. MM is the guarantor.

## Figures and Tables

**Appendix Table 1:** REFER patient characteristics.

Clinical parameters *n* (%)	Heart failure (*n* = 104)	No heart failure (*n* = 200)
Patients with previous MI with NT-proBNP levels < 125 pg/ml	1 (1.0)	10 (5.0)
Patients with previous MI with NT-proBNP levels greater than or equal to 125 pg/ml	16 (15.4)	7 (3.5)
Patients with basal crepitations without previous MI with NT-proBNP levels < 125 pg/ml	0 (0.0)	5 (2.5)
Patients with basal crepitations without previous MI with NT-proBNP levels between 125 pg/ml and 399 pg/ml	0 (0.0)	4 (2.0)
Patients with basal crepitations without previous MI with NT-proBNP levels equal to or above 400 pg/ml	3 (2.9)	2 (1.0)
Male patients with ankle oedema without basal crepitations or previous MI with NT-proBNP < 125 pg/ml	0 (0.0)	22 (11.0)
Male patients with ankle oedema without basal crepitations or previous MI with NT-proBNP levels between 125 pg/ml and 399 pg/ml	4 (3.8)	17 (8.5)
Male patients with ankle oedema without basal crepitations or previous MI with NT-proBNP levels equal to or above 400 pg/ml	22 (21.2)	5(2.5)
Female patients without ankle oedema without the MICE criteria with NT-proBNP levels < 125 pg/ml	0 (0.0)	9 (4.5)
Female patients without ankle oedema without the MICE criteria with NT-proBNP levels between 125 pg/ml and 399 pg/ml	0 (0.0)	6 (3.0)
Female patients without ankle oedema without the MICE criteria with NT-proBNP levels between 400 pg/ml and 619 pg/ml	0 (0.0)	2 (1.0)
Female patients without ankle oedema without the MICE criteria with NT-proBNP levels between 620 pg/ml and 1059 pg/ml	1 (1.0)	0 (0.0)
Female patients without ankle oedema without the MICE criteria with NT-proBNP levels equal to or above 1060 pg/ml	0 (0.0)	0 (0.0)
Female patients with ankle oedema without the MICE criteria with NT-proBNP levels < 125 pg/ml	4 (3.8)	43 (21.5)
Female patients with ankle oedema without the MICE criteria with NT-proBNP levels between 125 pg/ml and 189 pg/ml	3 (2.9)	15 (6.5)
Female patients with ankle oedema without the MICE criteria with NT-proBNP levels between 190 pg/ml and 399 pg/ml	4 (3.8)	31 (15.5)
Female patients with ankle oedema without the MICE criteria with NT-proBNP levels between 400 pg/ml and 519 pg/ml	4 (3.8)	5 (2.5)
Female patients with ankle oedema without the MICE criteria with NT-proBNP levels equal to or above 520 pg/ml	30 (28.8)	0 (0.0)
Male patients without ankle oedema without the MICE criteria with NT-proBNP levels < 125 pg/ml	1 (1.0)	9 (4.5)
Male patients without ankle oedema without the MICE criteria with NT-proBNP levels between 125 pg/ml and 389 pg/ml	2 (2.0)	7 (3.5)
Male patients without ankle oedema without the MICE criteria with NT-proBNP levels between 390 pg/ml and 399 pg/ml	1 (1.0)	0 (0.0)
Male patients without ankle oedema without the MICE criteria with NT-proBNP levels between 400 pg/ml and 659 pg/ml	2 (2.0)	0 (0.0)
Male patients without ankle oedema without the MICE criteria with NT-proBNP equal to or above 660 pg/ml	6 (5.8)	1 (0.50)

MI, myocardial infarction.

**Appendix Table 2:** Estimated undiscounted survival probabilities for male patients with heart failure on and off therapy.

Years	Untreated	ACEI	BB	Treated early	Treated late
0	1.00	1.00	1.00	1.00	1.00
0.25	0.73	0.77	0.79	0.77	0.73
0.5	0.68	0.72	0.75	0.72	0.68
1	0.57	0.62	0.66	0.62	0.59
2	0.46	0.52	0.57	0.52	0.49
5	0.25	0.29	0.36	0.31	0.29
10	0.11	0.13	0.20	0.15	0.14
11	0.08	0.10	0.16	0.12	0.11
12	0.05	0.07	0.12	0.08	0.07
13	0.03	0.03	0.07	0.04	0.04
14	0.00	0.00	0.02	0.01	0.00
15	0.00	0.00	0.00	0.00	0.00

**Appendix Table 3:** Estimated undiscounted survival probabilities for female patients with heart failure on and off therapy

Years	Untreated	ACEI	BB	Treated early	Treated late
0	1.00	1.00	1.00	1.00	1.00
0.25	0.72	0.76	0.79	0.76	0.72
0.5	0.69	0.74	0.77	0.74	0.69
1	0.64	0.69	0.72	0.69	0.65
2	0.56	0.61	0.65	0.61	0.58
5	0.38	0.43	0.49	0.44	0.41
10	0.21	0.25	0.32	0.26	0.25
11	0.18	0.21	0.28	0.23	0.21
12	0.14	0.17	0.24	0.19	0.17
13	0.11	0.13	0.20	0.15	0.14
14	0.07	0.09	0.15	0.11	0.10
15	0.04	0.05	0.10	0.06	0.06
16	0.01	0.01	0.02	0.01	0.01
17	0.00	0.00	0.00	0.00	0.00
